# A Maze Matrix-Based Secret Image Sharing Scheme with Cheater Detection

**DOI:** 10.3390/s20133802

**Published:** 2020-07-07

**Authors:** Ching-Chun Chang, Ji-Hwei Horng, Chia-Shou Shih, Chin-Chen Chang

**Affiliations:** 1 Department of Electronic Engineering, Tsinghua University, Beijing 100084, China; c.c.chang.phd@gmail.com; 2 Department of Electronic Engineering, National Quemoy University, Kinmen 89250, Taiwan; 3 Department of Information Engineering and Computer Science, Feng Chia University, Taichung 40724, Taiwan; jsfcu1129@gmail.com (C.-S.S.); ccc@o365.fcu.edu.tw (C.-C.C.); 4 School of Computer Science and Technology, Hangzhou Dianzi University, Hangzhou 310018, China

**Keywords:** secret image sharing, maze matrix, cheat detection, cheater identification

## Abstract

Secret image sharing is a technique for sharing a secret message in such a fashion that stego image shadows are generated and distributed to individual participants. Without the complete set of shadows shared among all participants, the secret could not be deciphered. This technique may serve as a crucial means for protecting private data in massive Internet of things applications. This can be realized by distributing the stego image shadows to different devices on the Internet so that only the ones who are authorized to access these devices can extract the secret message. In this paper, we proposed a secret image sharing scheme based on a novel maze matrix. A pair of image shadows were produced by hiding secret data into two distinct cover images under the guidance of the maze matrix. A two-layered cheat detection mechanism was devised based on the special characteristics of the proposed maze matrix. In addition to the conventional joint cheating detection, the proposed scheme was able to identify the tampered shadow presented by a cheater without the information from other shadows. Furthermore, in order to improve time efficiency, we derived a pair of Lagrange polynomials to compute the exact pixel values of the shadow images instead of resorting to time-consuming and computationally expensive conventional searching strategies. Experimental results demonstrated the effectiveness and efficiency of the proposed secret sharing scheme and cheat detection mechanism.

## 1. Introduction

Massive Internet of things (Massive IoT) involves an immense number of devices that require to be connected reliably and gigantic loads of data that need to travel safely through the Internet. With the growing public concerns over Internet privacy and security, there is an urgent appeal for research into secure communications in massive IoT. Pioneering works include the aggregate-signcryption [1], decentralized blockchain [2], FORGE system [3], and chaotic maps [4]. In this paper, we address this issue with a novel approach based on secret image sharing.

We propose to conceal the private data into a pair of image shadows and transmit them to separate devices over public networks. An authorized recipient should be able to access the image shadows stored on the separate devices and retrieve the private data via low-cost computations. The core component of the proposed secret image sharing scheme is the maze matrix, which belongs to a group of reference matrices originating from steganographic methods.

Steganography is the art and science of hiding information. It can be used to protect secret information by concealing it into cover images. These techniques can be broadly categorized into the transformed domain [5,6,7,8,9] and the spatial domain [10,11,12,13,14,15,16,17] methodologies. For the former class of methods, some commonly used transformations are the discrete cosine transform (DCT) [5,6], vector quantization (VQ) [7], and absolute moment block truncation coding (AMBTC) [8,9]. As for the latter class of methods, reference matrix-based algorithms have proved to be efficient in terms of the distortion versus capacity tradeoff. Common magic matrix-based steganographic schemes include the exploiting modified direction (EMD) [10,11], the turtle shell [12], the octagon-shaped shell [13], and the Sudoku [14,15,16,17] schemes.

Another closely related research stream is visual cryptography, which was first proposed by Naor and Shamir [18]. Typical visual cryptography schemes encrypt a secret image by breaking it up into n shares of obfuscated meaningless images, which are then printed onto separate transparencies. When k out of n transparencies are stacked and overlaid, the secret image will appear and become recognizable, where k is a pre-defined threshold. Methods of visual cryptography has constantly evolved, and the later developments contrived to produce shares in such a form that they themselves are images with meaningful contents [19,20,21,22].

A significant visual cryptography (SVC) [23] was recently proposed to securely transfer real-time images without compromising the visual quality. In the author’s scheme, random share values are hidden in a cover image by LSB embedding. The signific secret image with induced errors can be revealed using a (k, n) SVC scheme, while the exact secret image can be revealed using an (n, n) scheme. However, this scheme is not capable of detecting cheaters.

As a notable improvement, a verifiable secret sharing scheme with combiner verification and cheater identification [24] was recently developed. Its share generation and secret reconstruction mechanisms were based on the polynomial interpolation technique invented by Shamir [25]. Its combiner verification and cheater identification were realized via a pre-shared key and a verifier code generated from the combiner’s ID and password.

A recent development by Liu et al. [26] demonstrated that it is possible to identify the tampered shadows by restricting the use of elements at certain locations of the reference matrix and checking justness of the mapped elements in the secret extracting process. Through this mechanism, dishonest behaviors can be detected without the help of a pre-shared secret key or a password system.

In this paper, we proposed a novel secret image sharing scheme for massive IoT applications. The image shadows were generated under the guidance of the maze matrix. By leveraging the special characteristics of the maze matrix, we were able to inspect whether cheating behaviors took place. A two-layered cheat detection mechanism was devised. A joint cheat detection can discover cheating behaviors and a blind cheater identification can trace which shadow is inauthentic.

The proposed scheme shares the same merits as Liu et al.’s scheme, as that no pre-shared secret key or password system is required. In addition to this, the proposed maze matrix was explicitly designed to enable the scheme to detect cheats under the paradigm of secret sharing. Moreover, we formulated a pair of Lagrange polynomials to compute the exact pixel values of the shadow images rather than adopting time-consuming and computationally expensive conventional searching strategies. As a consequence, the time efficiency of the proposed share construction algorithm can be dramatically improved.

This remainder of this paper is organized as follows. Section 2 reviews a state-of-the-art secret image sharing scheme. Section 3 presents the proposed secret image sharing scheme based on maze matrix and the two-layered cheat detection mechanism. Experimental results and performance comparisons are shown in Section 4. This paper is concluded in Section 5.

## 2. Related Work

In this section, we briefly review the secret image sharing scheme proposed by Liu et al. [26] with a discussion of its merits and demerits. Our proposed scheme was based on the similar framework and is introduced in the next section.

**Figure 1:**
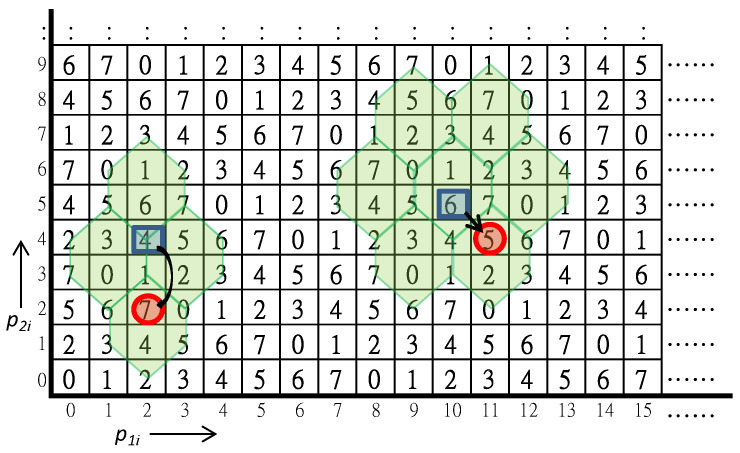
The turtle shell matrix for secret image sharing scheme.

The secret image sharing scheme proposed by Liu et al. [26] allows a dealer to share secret message into two different meaningful images. It adopts the turtle shell matrix $M(p_{1i},p_{2i}),$ proposed by Chang et al. [12], to guide the embedding of secret message, as shown in Figure 1. Before constructing secret shares of shadow images, the binary stream of secret message is converted to 8-ary secret set $S=\left\{sg_{k}|k=1,2,\ldots ,n\right\}$. The pixels of two distinct grayscale cover images with size H×W are rearranged into $C_{1}=\left\{p_{1i}|i=1,2,\ldots ,H\times W\right\}$ and $C_{2}=\left\{p_{2i}|i=1,2,\ldots ,H\times W\right\}$. Each pair of pixels $\left(p_{1i},p_{2i}\right)$ is used to embed a secret digit $sg_{k}$ in a way like conventional reference matrix-based data hiding scheme.

For the purpose of cheating detection, the elements in the reference matrix are classified into back elements and edge elements. As implied by the name, an edge element is an element located on the common edges of adjacent hexagons. On the contrary, a back element is located inside a single hexagon. The embedding rules are as follows. The cover pixel pair $\left(p_{1i},p_{2i}\right)$ is applied to locate a reference element in the matrix first. For an edge reference element, the rocket-shaped turtle shells as shown in the figure are the candidates of embedding, while the flower-shaped turtle shells are the candidates for a back-reference element. By searching the candidates to find the nearest back element that $M\left(p_{1i}^{\prime },p_{2i}^{\prime }\right)=sg_{k}$, the obtained pixels $\left(p_{1i}^{\prime },p_{2i}^{\prime }\right)$ are recorded to the image shadows. After all secret digits are embedded, the shadow images $S_{1}=\left\{p_{1i}^{\prime }|i=1,2,\ldots ,H\times W\right\}$ and $S_{2}=\left\{p_{2i}^{\prime }|i=1,2,\ldots ,H\times W\right\}$ are constructed. By restricting the embedding candidates to the back elements only, the cheating event can be detected while the shadow pixel pair $\left(p_{1i}^{\prime },p_{2i}^{\prime }\right)$ is mapped to an edge element $M\left(p_{1i}^{\prime },p_{2i}^{\prime }\right)$.

Two typical examples of data hiding are illustrated in Figure 1. In the first example, the cover pixel pair is $(p_{1i},p_{2i})=\left(2,4\right)$ and the secret digit is $sg_{k}=7$. First, the cover pixel pair (2,4) is mapped to the edge reference element M(2,4). By searching its associated rocket-shaped candidate turtle shells, the only matched back element is $M\left(2,2\right)=7=sg_{k}$. The recorded shadow pixels are therefore $\left(p_{1i}^{\prime },p_{2i}^{\prime }\right)=\left(2,2\right)$. Although M(3,5) and M(0,3) are also matched with the secret digit, they are not back elements and thus conflict with the embedding rule.

The second example uses $(p_{1i},p_{2i})=\left(10,5\right)$ and $sg_{k}=5$ as inputs. The reference element M(10,5) is a back element, therefore the candidates of embedding are the flower-shaped turtle shells shown in the figure. The matched candidates M(9,5) and M(12,7) are edge elements and excluded. Two legal candidates are M(11,4) and M(9,8). The nearest matched back element M(11,4) is the final solution and the shadow pixels are given by $\left(p_{1i}^{\prime },p_{2i}^{\prime }\right)=\left(11,4\right)$.

To extract secret data, both shares of the image shadows should be obtained from the participants. The corresponding pair of pixels from the two shadows is mapped to the secret digit through the guidance of the turtle shell matrix. In case an edge element is mapped, we can conclude someone is cheating. The exact cheater can only be identified by a faithful participant. To overcome this weak point, we propose a new scheme in the following section.

## 3. The Proposed Secret Image Sharing Scheme

The proposed secret image sharing scheme was to convert two distinct cover images into a pair of shadow images through the guidance of a new proposed maze matrix. By cooperating the pair of shadow images occupied by two different participants, the embedded secret data could be extracted. In addition, a cheater detection mechanism was devised such that any cheating share of shadow images could be detected without help of the other share.

### 3.1. The Maze Matrix

**Figure 2:**
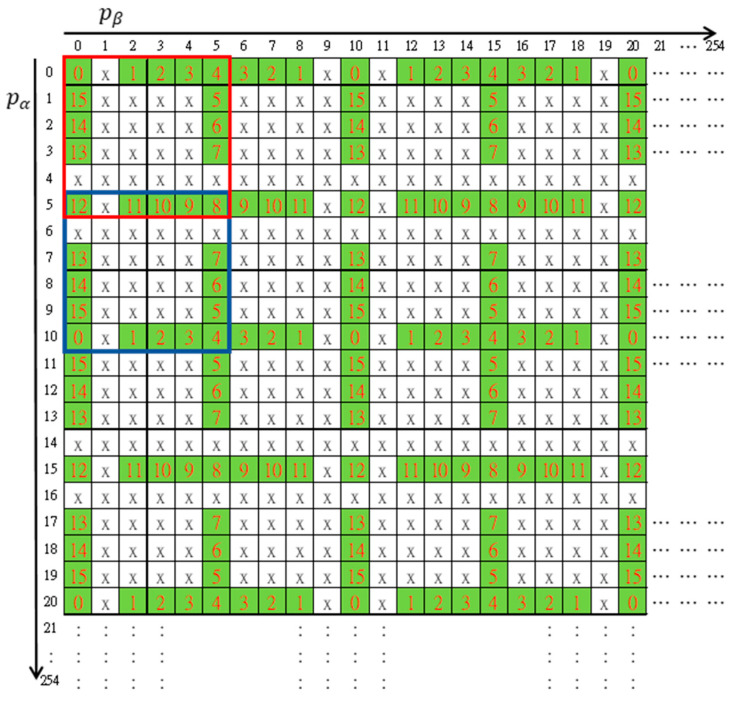
The maze matrix for secret image sharing scheme.

The maze matrix was constructed using a basic structure matrix of size 6×6 as enclosed by the red square shown in Figure 2. Distinct numbers in the radix-16 number system were arranged by circulating the outmost boundary of the region except for a horizontal and a vertical gap. Other elements were marked with ‘x’. By repeated mirroring operations, the rest of a 256×256 maze matrix was constructed. The first mirror matrix of the red basic structure to the $p_{\alpha }$ direction of axis was enclosed by a blue square in the figure. The resulting matrix $M\left(p_{\alpha },p_{\beta }\right)$ looks like a big maze map and was named the maze matrix.

### 3.2. The Data Embedding and Extraction Scheme

Following the same problem formulation as the turtle shells matrix-based secret image sharing scheme [26], we constructed a pair of image shadows using a pair of distinct cover images through the guidance of the proposed maze matrix.

Before constructing secret shares of shadow images, the binary stream of secret message was converted to 16-ary secret set $S=\left\{sg_{k}|k=1,2,\ldots ,n\right\}$. Pixels of two distinct grayscale cover images with size H×W are rearranged into $C_{1}=\left\{p_{1i}|i=1,2,\ldots ,H\times W\right\}$ and $C_{2}=\left\{p_{2i}|i=1,2,\ldots ,H\times W\right\}$.

Each pair of pixels $\left(p_{1i},p_{2i}\right)$ was used to embed a secret digit $sg_{k}$ through the guidance of the maze matrix. For a cover pixel pair $\left(p_{1i},p_{2i}\right)$, it was mapped to the maze matrix $M(p_{1i},p_{2i})$ first. Then, we searched the neighboring elements to find the nearest matched element $M\left(p_{1i}^{\prime },p_{2i}^{\prime }\right)=sg_{k}$ and record the shadow pixels $\left(p_{1i}^{\prime },p_{2i}^{\prime }\right)$ to the shadow images. After all secret digits were embedded, the shadow images $S_{1}=\left\{p_{1i}^{\prime }|i=1,2,\ldots ,H\times W\right\}$ and $S_{2}=\left\{p_{2i}^{\prime }|i=1,2,\ldots ,H\times W\right\}$ were constructed. Note that the elements marked with ‘x’ can never be the target element. This property will be applied to devise the cheating detection mechanism.

The data extraction process is rather simple: Collect the pair of image shadows provided by different participants and construct the same maze matrix as embedding. Then, consecutively extract secret digits by $sg_{k}=M\left(p_{1i}^{\prime },p_{2i}^{\prime }\right)$ until all secrets are extracted.

To detect cheating events, the ‘x’-marked forbidden zone is the key. Any pair of shadow pixels which maps to an ‘x’-marked element indicates someone is cheating. In addition, while the mapped element lies at a horizontal or vertical gap of the maze matrix, the exact cheater can be identified.

The detailed algorithms of the data embedding and extraction processes are discussed in the following subsections. In the last subsection, we discuss the cheater detection mechanism of the proposed secret image sharing scheme.

### 3.3. The Sshare Construction Algorithm

As described in the previous subsection, a secret digit is embedded by modifying the cover pixel pair to the target element through the guidance of maze matrix. However, the searching process is time-consuming. To improve the embedding efficiency, we devised a Lagrange polynomial to determine the target element of modification. Let $\left(p_{x},p_{y}\right)$ be the cover pixel pair in the range $0\leq p_{x}\leq 4,\;0\leq p_{y}\leq 4$. According to the maze matrix as shown in Figure 2, the target elements of modification for embedding different secret digits are listed in Table 1.

Let

(1) $$X=\left\{x_{0},x_{1},x_{2},\cdots ,x_{15}\right\}=\left\{0,0,0,0,0,1,2,3,5,5,5,5,5,3,2,1\right\},$$

(2) $$Y=\left\{y_{0},y_{1},y_{2},\cdots ,y_{15}\right\}=\left\{0,2,3,4,5,5,5,5,5,4,3,2,0,0,0,0\right\}.$$

The modified pixel pair $\left(p_{x}^{\prime },p_{y}^{\prime }\right)$ can be represented by the Lagrange polynomial functions of $s_{j}$ as shown below:

(3) $$p_{x}^{\prime }\left(sg_{j}\right)=\sum_{r=0}^{15}x_{r}\prod_{\begin{array}{c} k\neq r\\ k=0 \end{array}}^{15}\frac{\left(sg_{j}-k\right)}{\left(r-k\right)},$$

(4) $$p_{y}^{\prime }\left(sg_{j}\right)=\sum_{r=0}^{15}y_{r}\prod_{\begin{array}{c} k\neq r\\ k=0 \end{array}}^{15}\frac{\left(sg_{j}-k\right)}{\left(r-k\right)}.$$

By leveraging the periodic property of the maze matrix, we modulated a reference element $M\left(p_{\alpha },p_{\beta }\right)$ to the fundamental period of $M\left(p_{x},p_{y}\right)$, $0\leq p_{x}\leq 9,\;0\leq p_{y}\leq 9$. Then, the fundamental period was further divided into four reflective symmetric parts. According to the secret digit $s_{j}$ to be embedded, a quasi-target element $M\left(p_{x}^{\prime }\left(sg_{j}\right),p_{y}^{\prime }\left(sg_{j}\right)\right)$ can be obtained. By reflection and backward modulation, the target element $M\left(p_{\alpha }^{\prime },p_{\beta }^{\prime }\right)$ can be determined. The detailed algorithm is summarized as follows.

The construction of image shadows:

Input: Cover images $C_{1}$ and $C_{2}$, secret message S

Output: Image shadows $S_{1}$ and $S_{2}$

Step 1. Arrange the cover images into two separate pixel streams and convert the secret message to 16-ary secret digits.

(5) $$C_{1}=\left\{p_{1i}|i=1,2,\ldots ,H\times W\right\},$$

(6) $$C_{2}=\left\{p_{2i}|i=1,2,\ldots ,H\times W\right\},$$

where H×W is the image size.

(7) $$S=\left\{sg_{k}|k=1,2,\ldots ,n\right\},$$

where n is the total number of digits.

Step 2. Retrieve a cover pixel pair $\left(p_{1i},p_{2i}\right)$ and let

(8) $$\left(p_{\alpha },p_{\beta }\right)=\{\begin{array}{cc} (p_{1i},p_{2i}), & \mathrm{for}\;i\;\mathrm{is}\;\mathrm{odd},\\ (p_{2i},p_{1i}), & \mathrm{for}\;i\;\mathrm{is}\;\mathrm{even}. \end{array}$$

Step 3. Modulate the pixel values to the fundamental period.

(9) $$p_{x}=mod\left(p_{\alpha },\;10\right),$$

(10) $$p_{y}=mod\left(p_{\beta },\;10\right).$$

(11) $$M=\lfloor \frac{p_{\alpha }}{10}\rfloor ,$$

(12) $$N=\lfloor \frac{p_{\beta }}{10}\rfloor .$$

Step 4. Using the Lagrange polynomial defined as Equations (1) to (4), determine the target element of modification.

For $0\leq p_{x}\leq 4\;\mathrm{and}\;0\leq p_{y}\leq 4$,

(13) $$p_{\alpha }^{\prime }=p_{x}^{\prime }\left(sg_{j}\right)+10\times M;$$

(14) $$p_{\beta }^{\prime }=p_{y}^{\prime }\left(sg_{j}\right)+10\times N.$$

For $5\leq p_{x}\leq 9\;\mathrm{and}\;0\leq p_{y}\leq 4$,

(15) $$p_{\alpha }^{\prime }=\left[10-p_{x}^{\prime }\left(sg_{j}\right)\right]+10\times M;$$

(16) $$p_{\beta }^{\prime }=p_{y}^{\prime }\left(sg_{j}\right)+10\times N.$$

For $0\leq p_{x}\leq 4\;\mathrm{and}\;5\leq p_{y}\leq 9$,

(17) $$p_{\alpha }^{\prime }=p_{x}^{\prime }\left(sg_{j}\right)+10\times M;$$

(18) $$p_{\beta }^{\prime }=\left[10-p_{y}^{\prime }\left(sg_{j}\right)\right]+10\times N.$$

For $5\leq p_{x}\leq 9\;\mathrm{and}\;5\leq p_{y}\leq 9$,

(19) $$p_{\alpha }^{\prime }=\left[10-p_{x}^{\prime }\left(sg_{j}\right)\right]+10\times M;$$

(20) $$p_{\beta }^{\prime }=\left[10-p_{y}^{\prime }\left(sg_{j}\right)\right]+10\times N.$$

Step 5. Record the shadow pixels.

(21) $$\left(p_{1i}^{\prime },p_{2i}^{\prime }\right)=\{\begin{array}{cc} \left(p_{\alpha }^{\prime },p_{\beta }^{\prime }\right), & \mathrm{for}\;i\;\mathrm{is}\;\mathrm{odd},\\ \left(p_{\beta }^{\prime },p_{\alpha }^{\prime }\right), & \mathrm{for}\;i\;\mathrm{is}\;\mathrm{even}. \end{array}$$

Step 6. Repeat Step 2 to 5 until all secret digits are embedded.

Step 7. Output the pair of image shadows.

(22) $$S_{1}=\left\{p_{1i}^{\prime }|i=1,2,\ldots ,H\times W\right\};$$

(23) $$S_{2}=\left\{p_{2i}^{\prime }|i=1,2,\ldots ,H\times W\right\}.$$

Note that there are many gaps at $\mathrm{mod}\left(p_{\alpha },10\right)=4,6$ and $\mathrm{mod}\left(p_{\beta },10\right)=1,9$ of the maze matrix. Using the conventional fixed assignment of $\left(p_{\alpha },p_{\beta }\right)=(p_{1i},p_{2i})$, the resulting $\left(p_{\alpha }^{\prime },p_{\beta }^{\prime }\right)$ will lack the gapped pixel values. This may draw the eavesdropper’s attention. To prevent the vacuums of pixel value, we alternatively assigned $\left(p_{\alpha },p_{\beta }\right)$ with $\left(p_{1i},p_{2i}\right)$ and $\left(p_{2i},p_{1i}\right)$ in Step 2 and switched back in Step 5 coordinately. The asymmetric gapping of maze matrix in the $p_{\alpha }$ and $p_{\beta }$ directions made it possible to cover the gaps by leveraging the alternating assignment.

We provide two examples to demonstrate the operation of embedding process. Assume the first cover pixel pair is $(p_{11},p_{21})=\left(83,61\right)$ and the secret digit to be embedded is $sg_{1}=5$. Following the steps of embedding algorithm gives $\left(p_{\alpha },p_{\beta }\right)=\left(83,61\right)$, $\left(p_{x},p_{y}\right)=\left(3,1\right)$, (M,N)=(8,6), $(p_{x}^{\prime }\left(5\right),p_{y}^{\prime }\left(5\right))=\left(1,5\right)$, and $\left(p_{11}^{\prime },p_{21}^{\prime }\right)=\left(p_{\alpha }^{\prime },p_{\beta }^{\prime }\right)=\left(1+8\times 10,5+6\times 10\right)=\left(81,65\right)$. Let the second cover pixel pair and the second secret digit be $(p_{12},p_{22})=\left(83,66\right)$ and $sg_{2}=14$. Following the same calculation gives $\left(p_{\alpha },p_{\beta }\right)=(p_{22},p_{12})=\left(66,83\right)$, $\left(p_{x},p_{y}\right)=\left(6,3\right)$, (M,N)=(6,8), $(p_{x}^{\prime }\left(14\right),p_{y}^{\prime }\left(14\right))=\left(2,0\right)$, and $\left(p_{\alpha }^{\prime },p_{\beta }^{\prime }\right)=\left(\left(10-2\right)+6\times 10,0+8\times 10\right)=\left(68,80\right)$, and $\left(p_{12}^{\prime },p_{22}^{\prime }\right)=\left(p_{\beta }^{\prime },p_{\alpha }^{\prime }\right)=\left(80,68\right)$.

### 3.4. The Data Extraction Algorithm

The secret message can be extracted only through cooperation of the two shadow image owners. The secret data can be extracted by pairing the pixels from the two image shadows and applying each pixel pair to retrieve a 16-ary secret digit through the guidance of maze matrix. The 16-ary secret digits can be converted back to the binary secret stream if necessary. The data extraction algorithm is provided as follows.

The data extraction algorithm:

Input: image shadows $S_{1}$ and $S_{2}$

Output: secret message S

Step 1. Arrange the image shadows into two separate pixel streams.

(24) $$S_{1}=\left\{p_{1i}^{\prime }|i=1,2,\ldots ,H\times W\right\};$$

(25) $$S_{2}=\left\{p_{2i}^{\prime }|i=1,2,\ldots ,H\times W\right\},$$

where H×W is the image size.

Step 2. Construct the fundamental period of maze matrix $M\left(p_{x},p_{y}\right)$, $0\leq p_{x}\leq 9,\;0\leq p_{y}\leq 9$ as shown in Figure 2.

Step 3. Retrieve a shadow pixel pair $\left(p_{1i}^{\prime },p_{2i}^{\prime }\right)$ and let

(26) $$\left(p_{\alpha },p_{\beta }\right)=\{\begin{array}{cc} \left(p_{1i}^{\prime },p_{2i}^{\prime }\right), & \mathrm{for}\;i\;\mathrm{is}\;\mathrm{odd},\\ (p_{2i}^{\prime },p_{1i}^{\prime }), & \mathrm{for}\;i\;\mathrm{is}\;\mathrm{even}. \end{array}$$

Step 4. Extract the secret digit $sg_{j}$ and record to S.

(27) $$sg_{j}=M\left(mod\left(p_{\alpha },\;10\right),mod\left(p_{\beta },\;10\right)\right).$$

Step 5. Repeat Step 3 and 4 until all secret digits are extracted.

Step 6. Convert the 16-ary secret digits back to the binary secret stream.

Now, we apply the embedding results in the previous subsection $\left(p_{11}^{\prime },p_{21}^{\prime }\right)=\left(81,65\right)$ and $\left(p_{12}^{\prime },p_{22}^{\prime }\right)=\left(80,68\right)$ as examples. For the first shadow pixel pair, the secret digit can be retrieved by directly calculating Equation (27), i.e., $sg_{1}=M\left(mod\left(81,\;10\right),\;mod\left(65,\;10\right)\right)=M\left(1,5\right)=5$. For the second pixel pair, the pixels should be swapped according to Equation (26), i.e., $\left(p_{\alpha },p_{\beta }\right)=\left(68,80\right)$. Then, calculate Equation (27),, i.e., $sg_{2}=M\left(mod\left(68,\;10\right),\;mod\left(80,\;10\right)\right)=M\left(8,0\right)=14$. Both secret digits coincided with the embedded ones.

### 3.5. The Cheat Event Detection and Cheater Detection Mechanism

The most creative part of our secret sharing scheme was the cheater detection mechanism. Referring to Figure 2, the ‘x’-marked elements in the maze matrix were the traps. Any pair of shadow pixels which maps to an ‘x’-marked element was illegal and served as key information for cheat event detection. The algorithm is given as follows.

The cheat detection algorithm:

Input: image shadows $S_{1}$ and $S_{2}$

Output: cheating pixel pairs F, cheating pixels $F_{1}$ and $F_{2}$

Step 1. Arrange the image shadows into two separate pixel streams.

(28) $$S_{1}=\left\{p_{1i}^{\prime }|i=1,2,\ldots ,H\times W\right\};$$

(29) $$S_{2}=\left\{p_{2i}^{\prime }|i=1,2,\ldots ,H\times W\right\},$$

where H×W is the image size.

Step 2. Construct the fundamental period of maze matrix $M\left(p_{x},p_{y}\right)$, $0\leq p_{x}\leq 9,\;0\leq p_{y}\leq 9$ as shown in Figure 2.

Step 3. Retrieve a shadow pixel pair $\left(p_{1i}^{\prime },p_{2i}^{\prime }\right)$ and let

(30) $$\left(p_{\alpha },p_{\beta }\right)=\{\begin{array}{cc} \left(p_{1i}^{\prime },p_{2i}^{\prime }\right), & \mathrm{for}\;i\;\mathrm{is}\;\mathrm{odd},\\ (p_{2i}^{\prime },p_{1i}^{\prime }), & \mathrm{for}\;i\;\mathrm{is}\;\mathrm{even}. \end{array}$$

Step 4. Detect cheating pixel pairs and individual cheating pixels.

if $M\left(mod\left(p_{\alpha },\;10\right),mod\left(p_{\beta },\;10\right)\right)=\prime x\prime$,

  record i to F;

  if $mod\left(p_{\alpha },\;10\right)=4\;\mathrm{or}\;6$,

    record i to $F_{1}$ for i is odd; record i to $F_{2}$ for i is even.

  end

  if $mod\left(p_{\beta },\;10\right)=1\;\mathrm{or}\;9$,

    record i to $F_{2}$ for i is odd; record i to $F_{1}$ for i is even.

  end

end

Step 5. Repeat Step 3 and 4, until all pixel pairs are checked.

The cheat detection included two layers. The outer layer was a joint cheat event detection. The shadow pixel pair was mapped to the maze matrix and check the legality. If an ‘x’-marked element was mapped, the index i of the pixel pair was recorded to F. Under such circumstances, we could conclude that a cheat event was detected. The exact cheater could only be determined by a faithful participant. The inner layer was a blind cheater detection. We checked whether the mapped element was located at a gap. If it was located at a horizontal gap, $p_{\alpha }$ was a tampered pixel no matter what value $p_{\beta }$ is, because it was impossible to find a $p_{\beta }$ to make the pixel pair $\left(p_{\alpha },p_{\beta }\right)$ legal. For the same reason, a $p_{\beta }$ trapped in a vertical gap was a tampered pixel, and the participant who shared this shadow pixel was the cheater. The output sets $F_{1}$ and $F_{2}$ recorded the indices of tampered pixels from image shadows $S_{1}$ and $S_{2}$, respectively.

## 4. Experimental Results

**Figure 3:**
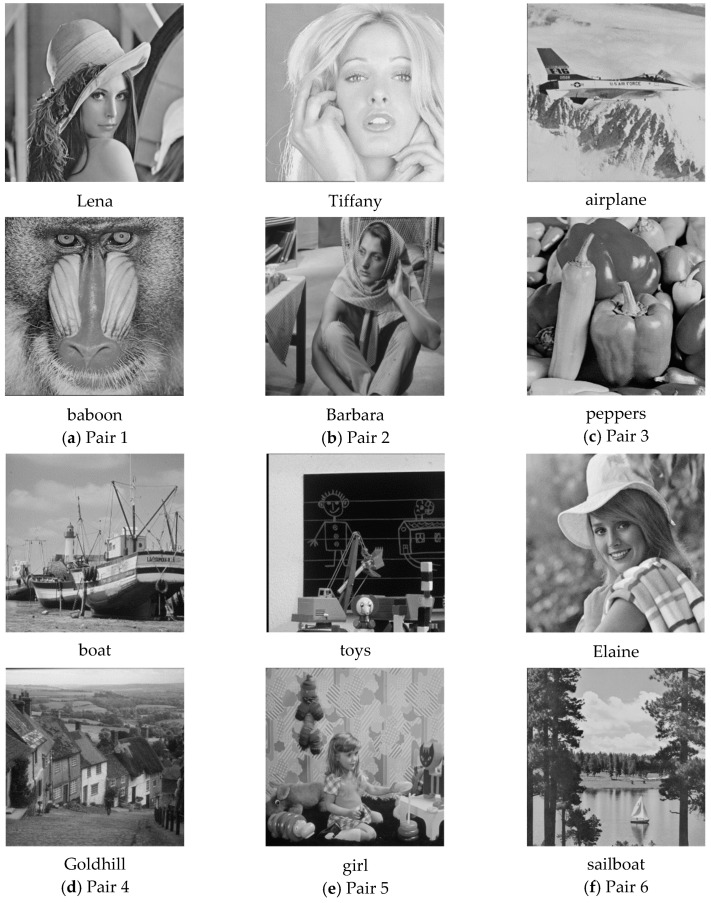
Six pairs of grayscale cover images.

**Figure 4:**
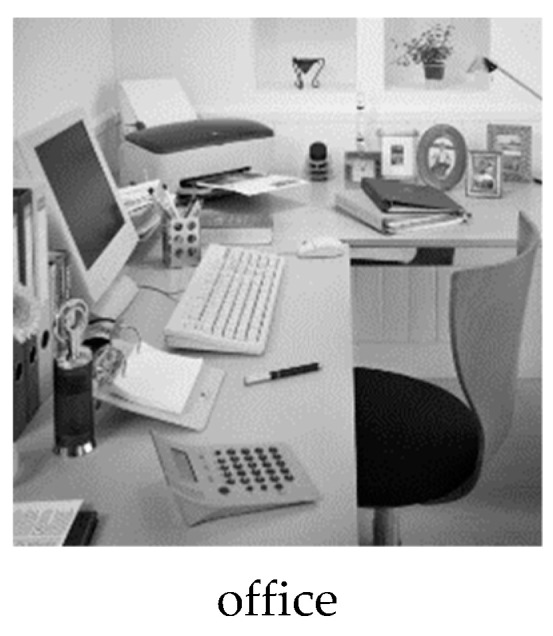
Secret image.

In this section, we give some experimental results to show the performance of the proposed secret image sharing scheme. Figure 3 shows six pairs of 512×512 grayscale cover images, including (a) Lena and baboon, (b) Tiffany and Barbara, (c) airplane and peppers, (d) boat and Goldhill, (e) toys and girl, and (f) Elaine and sailboat. According to the embedding capacity of the proposed scheme, we used a 362×362 grayscale secret image “office,” as shown in Figure 4. The embedding capacity of a cover image pair was 512×512×4=1,048,576 bits, while the secret image contained 362×362×8=1,048,352 bits of data. The whole secret image can be embedded into a cover image pair. The remaining capacity was filled with random generated data.

This section includes four subsections. In the first subsection, we demonstrate the applicability of the proposed share construction and data extraction scheme. The visual quality of the secret image shadows is also assessed. In the second subsection, we measure the detection ratio of tampered image regions. The effectiveness of cheat event detection and cheater detection are discussed. In the third subsection, the performance, including visual quality, hiding capacity, and cheat detection effectiveness, is compared with the Liu et al.’s scheme, which shares the same framework of secret image sharing scheme. Finally, the time efficiency of the new proposed share construction scheme is compared with conventional version in the last subsection.

### 4.1. Share Construction and Data Extraction

**Figure 5:**
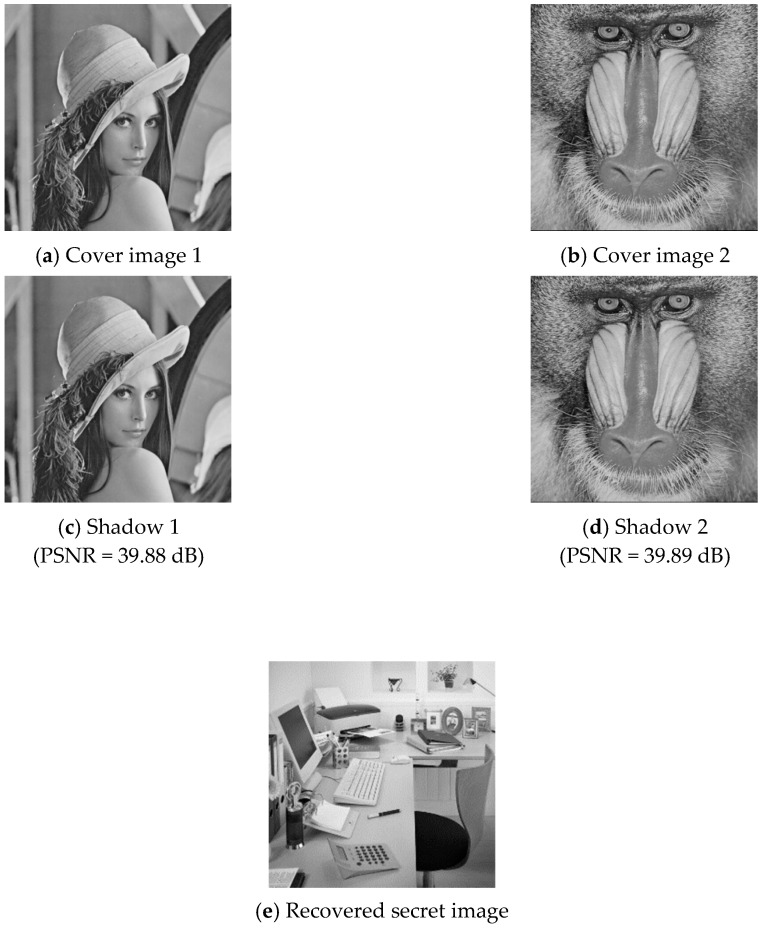
Experimental results of cover image pair 1.

**Figure 6:**
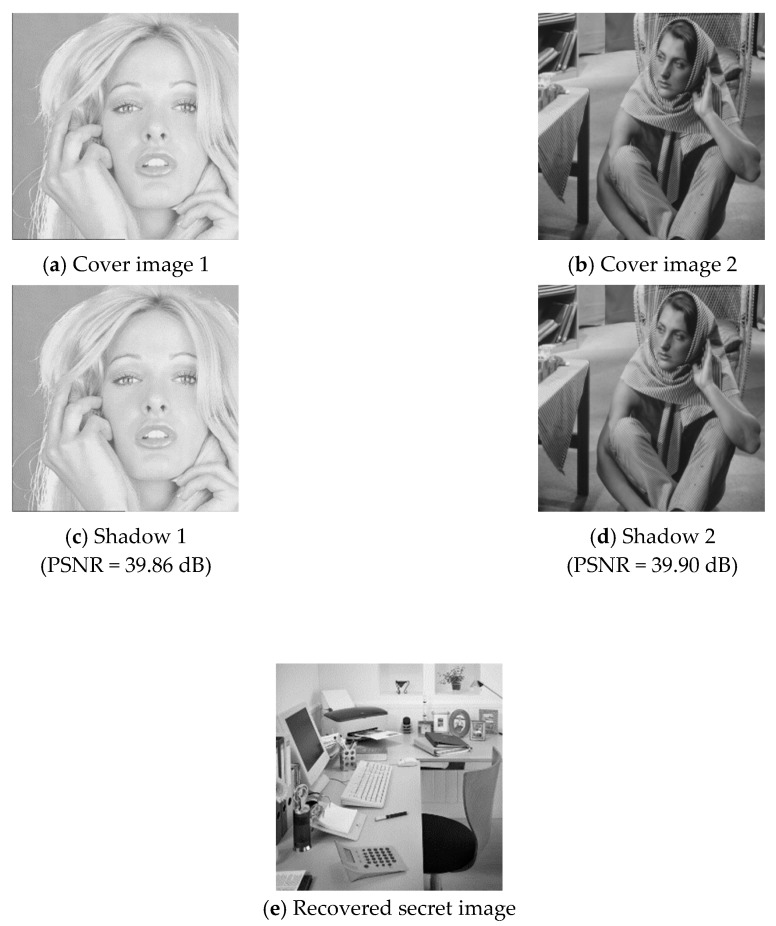
Experimental results of cover image pair 2.

To demonstrate the applicability of the proposed secret image sharing scheme, all six pairs of cover images were tested. Two examples of the experimental results are shown in Figure 5 and Figure 6, where (a) and (b) are the cover images, (c) and (d) are the shadow images, and (e) is the recovered secret image. As shown in the figures, the difference between a cover image and its corresponding shadow image cannot be distinguished by human eyes.

To evaluate the visual quality of the shadow images, we applied the peak-signal-to-noise ratio (PSNR), defined by

(31) $$PSNR=10\log_{10}\frac{255^{2}}{MSE}\left(\mathrm{dB}\right),$$

where MSE is the mean square error between the cover image $C_{k}$ and its corresponding shadow image $S_{k}$, defined by

(32) $$MSE=\frac{1}{H\times W}\sum_{i=1}^{H}\sum_{j=1}^{W}{\left(C_{k}\left(i,j\right)-S_{k}\left(i,j\right)\right)}^{2}.$$

The visual quality and embedding capacity for the six cover image pairs are listed in Table 2.

### 4.2. Cheat Event Detection and Cheater Detection

**Figure 7:**
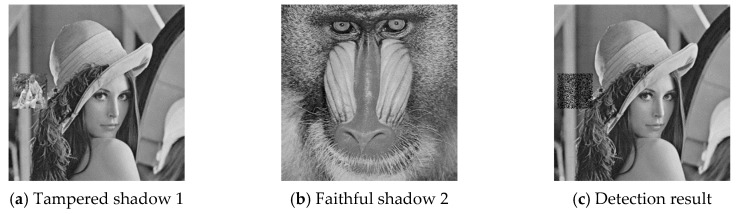
Joint cheat detection result 1.

**Figure 8:**
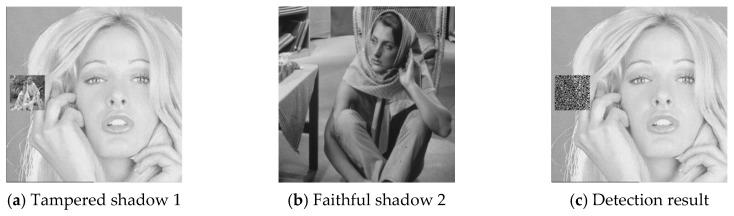
Joint cheat detection result 2.

**Figure 9:**
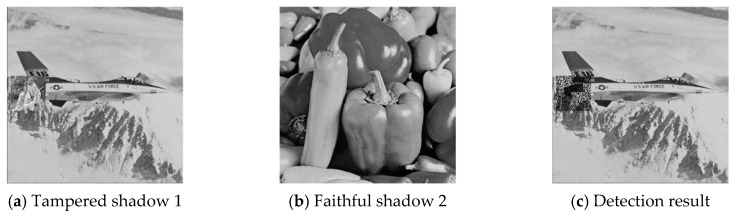
Joint cheat detection result 3.

**Figure 10:**
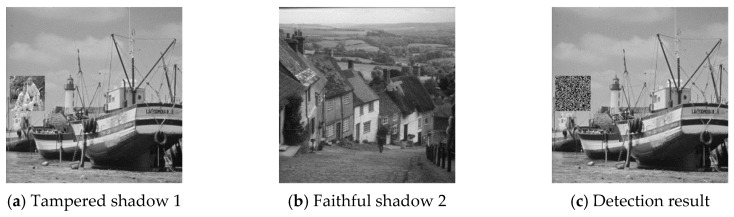
Joint cheat detection result 4.

The six pairs of image shadows were then applied to test the cheating detection mechanism. In each pair of shadows, shadow 1 was tampered by inserting a small image into a local region while shadow 2 was kept faithful. Four results of the six experiments are provided in Figure 7, Figure 8, Figure 9 and Figure 10, where (a) is the tampered shadow image 1, (b) is the faithful shadow image 2, and (c) is the result of joint detection. The detected cheat pixel pairs are illustrated by black pixels on the tampered shadow. The joint cheat detection ratio for the six test shadow pairs are listed in Table 3. In each test pair, the detection ratio was calculated by

(33) $$DR_{J}=\frac{N\left(F\right)}{N},$$

where N(F) is the number of total detected cheat pixel pairs and N is the number of tampered pixels, i.e., the total number of pixels in the inserted small image. As shown in the table, ${DR}_{J}$ of the joint cheat detection was around 0.42 and independent of the image features.

The blind cheater detection results for the six tampered shadows are listed in Table 4. The detection ratio for blind cheater detection is defined by

(34) $$DR_{B1}=\frac{N\left(F_{1}\right)}{N},$$

where $N\left(F_{1}\right)$ is the number of total detected pixel in shadow 1 by blind cheater detection and N is the number of tampered pixels, i.e., the total number of pixels in the inserted small image. As shown in the table, $DR_{B}$ of the blind cheater detection is around 0.20 and independent of the image features. Since the image shadow 2 was not tampered, the number of detected pixels $N\left(F_{2}\right)$ and thus $DR_{B2}$ are both zeros.

**Figure 11:**
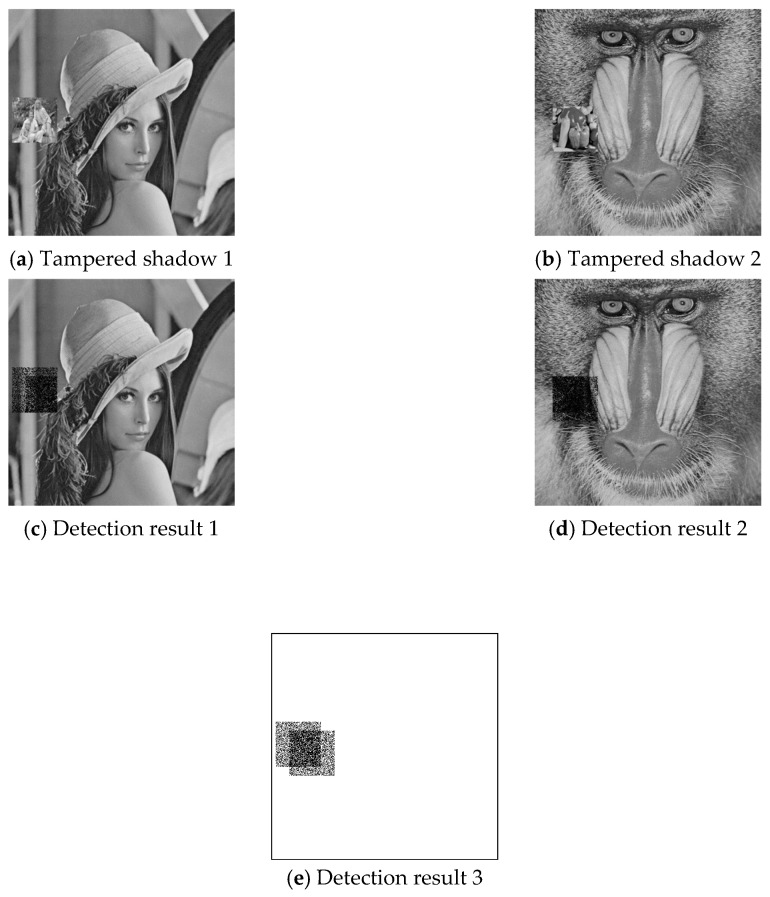
Joint cheating detection for combinatorial tampered shadows: Results for shadow pair 1.

**Figure 12:**
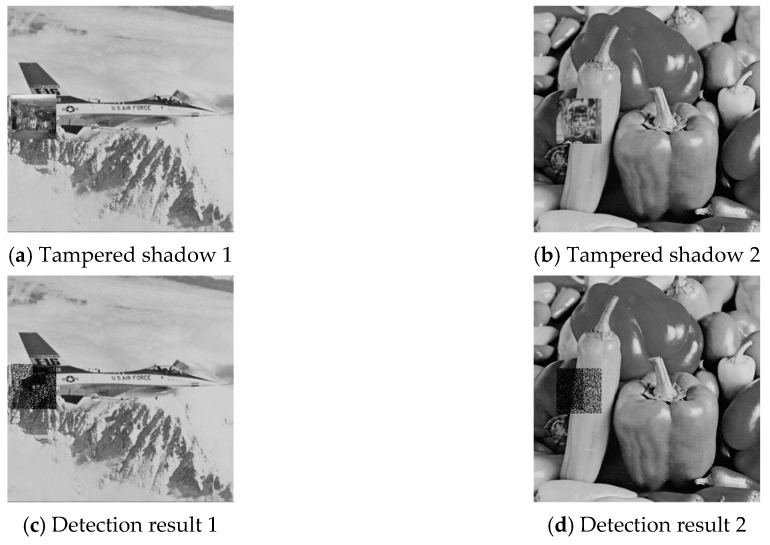

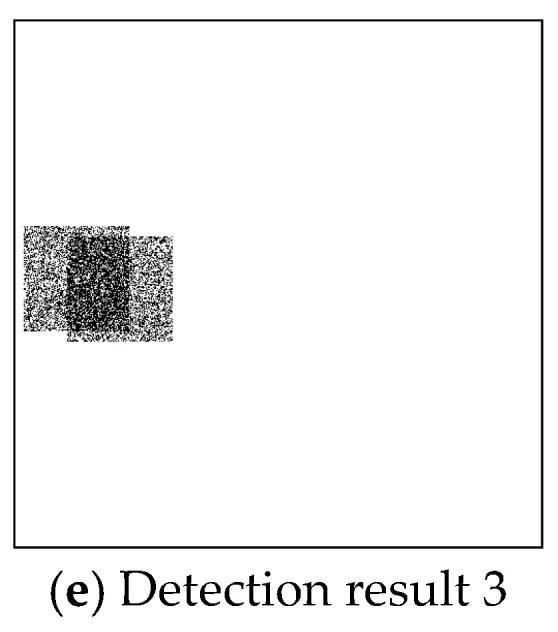
Joint cheating detection for combinatorial tampered shadows: Results for shadow pair 3.

To investigate the effect of combinatorial tampering, we further designed an experiment in which both image shadows were tampered with dis-aligned regions. Example results are given in Figure 11 and Figure 12, where (a) and (b) are the cover image pair, (c) and (d) are the detection results of joint cheat detection, and (e) illustrates the overview of total detected pixels. The experimental data for all six test shadow image pairs are listed in Table 5, where $DR_{1}$/$DR_{2}$ is the joint cheating detection ratio ($DR_{J}$) of the region that shadow 1/shadow 2 is tampered only; $DR_{1\cap 2}$ is the $DR_{J}$ of the region that both shadow1 and shadow 2 are tampered; $DR_{1\cup 2}$ is the $DR_{J}$ of the union tampered region. The joint cheating detection ratio ($DR_{1}$/$DR_{2}$) was around 43% for single tampered pixel pairs, while it was increased to 72% for combinatorial tampered pixel pairs ($DR_{1\cap 2}$). Both of the percentage numbers were independent of the image features since the proposed data hiding scheme was a uniform embedding scheme [27]. The detection ratio of the union region ($DR_{1\cup 2}$) depended on the percentage of overlapped region and was not an intrinsic characteristic of the proposed scheme.

### 4.3. Comparison with Liu et al.’s Scheme [26]

The comparison of the proposed maze matrix-based data hiding scheme with the turtle shell matrix-based scheme [26] is provided in Table 6. The new proposed scheme can hide four bits of secret data for each pair of cover pixels, while the turtle shell matrix-based scheme can hide only three bits for each pair. The EC given in the table was measured by bits per pixel pair, one from cover image 1 and the other from cover image 2. Due to different embedding capacity, the PSNR of the proposed scheme was slightly lower than the turtle shell scheme. However, the degradation of visual quality could not be recognized by human eyes.

The joint cheat detection ratio of the turtle shell scheme was 50% in both single tampered or combinatorial tampered cases. Although only the single tampered data was provided by the authors, the combinatorial tampered detection ratio can be analyzed easily. Since legal hiding locations are the back elements of turtle shells and such elements occupy 50% of the entire matrix, the theoretic cheating detection ratio was 50%. Our cheating detection mechanism outperformed the turtle shell scheme in combinatorial tampering, while the detection ratio was lower in single tampering.

The most creative part of the proposed scheme is the function of blind cheater detection. Without information of the other shadow, we detected 20% of tampered pixels in the shadow shared by a cheater. Meanwhile, the turtle shell scheme can only identify a cheater by a faithful participant.

### 4.4. Time Efficiency Evaluation

To assess the time efficiency of the proposed secret image sharing scheme, we listed the execution time required for the share construction program in Table 7 and the execution time for secret data extraction program in Table 8. The conventional reference matrix-based data hiding scheme and share construction scheme usually embed secret data by searching the nearest element that matches the intended secret digit and modify the pixel values accordingly. This type of searching procedures is often time-consuming. In this paper, a pair of Lagrange polynomials was derived to compute the coordinates of the matched element. Thus, the running time for share construction was drastically reduced. Referring to Table 7, up to 39% of execution time can be saved by leveraging the proposed approach. The execution time required for data extraction grogram is relatively short in comparison with the share construction program as shown in Table 8.

## 5. Conclusions

In this paper, we proposed a secret image sharing scheme based on a novel maze matrix. A pair of distinct cover images was used to carry secret data and a pair of shadow images was constructed under the guidance of the maze matrix. The secret data is extracted only if both authentic shadows are presented.

A two-layered cheat detection mechanism was devised to examine cheating behaviors as well as to ascertain the inauthentic shadow. In the outer cheat detection layer, the corresponding pair of pixels retrieved from the two shares was jointly used for detecting cheat events. The detection ratio was 43% for the cases in which single shadow was tampered and was 72% for the cases in which both shadows were tampered. In the inner blind cheater identification layer, the cheater’s image share could be spotted without the information from the other share. The detection ratio of tampered pixels was 20% for the blind cheater identification.

An additional merit of the proposed scheme is time efficiency. By computing the pixel values of the image shadows with Lagrange polynomials instead of conventional searching algorithms, the proposed approach can save up to 39% of program execution time. In view of the effectiveness and low power consumption of the proposed scheme, the outlook for integrating it with massive IoT systems as a data security module shall be positive.

In the future world where massive IoT environment is fully established, secret image sharing will no longer be restricted to share secrets among human participants. The image shadows produced by the dealer can be transmitted via different routes to devices located at different sites. The shadow production and secret extraction will be executed via APPs installed on smartphones of the dealer and receiver. Uploading and downloading image shadows through IoT links will permit secret data to be communicated securely without the use of a preshared key or password system.

## Figures and Tables

**Table 1:** Target elements of modification for different secret digits.

$sg_{j}$	$\left(p_{x}^{\prime },p_{y}^{\prime }\right)$	$sg_{j}$	$\left(p_{x}^{\prime },p_{y}^{\prime }\right)$	$sg_{j}$	$\left(p_{x}^{\prime },p_{y}^{\prime }\right)$	$sg_{j}$	$\left(p_{x}^{\prime },p_{y}^{\prime }\right)$
0	(0, 0)	4	(0, 5)	8	(5,5)	12	(5, 0)
1	(0, 2)	5	(1, 5)	9	(5, 4)	13	(3, 0)
2	(0, 3)	6	(2, 5)	10	(5, 3)	14	(2, 0)
3	(0, 4)	7	(3, 5)	11	(5, 2)	15	(1, 0)

**Table 2:** Experimental values of the proposed scheme.

	Cover Image 1	Cover Image 2	PSNR (dB)		Embedding Capacity (bits)
			Shadow 1	Shadow 2	
Pair 1	Lena	baboon	39.88	39.89	1,048,576
Pair 2	Tiffany	Barbara	39.86	39.90	1,048,576
Pair 3	airplane	peppers	39.87	39.88	1,048,576
Pair 4	boat	Goldhill	39.88	39.88	1,048,576
Pair 5	toys	girl	39.90	39.88	1,048,576
Pair 6	Elaine	sailboat	39.88	39.88	1,048,576

**Table 3:** Joint cheat detection ratio for the six shadow pairs.

Tampered Shadow	*DR_J_*
Lena	0.42
Tiffany	0.42
airplane	0.42
boat	0.42
toys	0.42
Elaine	0.42

**Table 4:** Blind cheater detection ratio for the six tampered shadows.

Tampered Shadow	*DR_B1_*
Lena	0.20
Tiffany	0.20
airplane	0.20
boat	0.20
toys	0.20
Elaine	0.20

**Table 5:** DR values for the six combinatorial tampered shadow pairs.

	Shadow 1	Shadow 2	$DR_{1}$	$DR_{2}$	$DR_{1\cap 2}$	$DR_{1\cup 2}$
Pair 1	Lana	baboon	0.43	0.43	0.72	0.52
Pair 2	Tiffany	Barbara	0.42	0.42	0.73	0.52
Pair 3	airplane	peppers	0.44	0.42	0.73	0.53
Pair 4	boat	Goldhill	0.43	0.43	0.71	0.52
Pair 5	toys	girl	0.43	0.43	0.71	0.53
Pair 6	Elaine	sailboat	0.43	0.43	0.72	0.53

**Table 6:** Comparison of the proposed maze matrix-based scheme with the turtle shell-based scheme.

Hiding Scheme	PSNR	EC	$DR_{JS}$	$DR_{JC}$	$DR_{B}$
Maze matrix	39.88	4	0.43	0.72	0.20
Turtle shell [26]	41.71	3	0.50	0.50	—

**Table 7:** Efficiency comparison of the proposed embedding scheme with conventional scheme.

Cover Images	Execution Time (sec)	
	Conventional Scheme	Proposed Scheme
Pair 1	0.1297	0.0692
Pair 2	0.1425	0.0747
Pair 3	0.1074	0.0737
Pair 4	0.1030	0.0703
Pair 5	0.1110	0.0708
Pair 6	0.1055	0.0709
**Average**	0.1165	0.0716

**Table 8:** Efficiency of the extraction scheme.

Stego Images	Execution Time (sec)
Pair 1	0.0366
Pair 2	0.0361
Pair 3	0.0382
Pair 4	0.0366
Pair 5	0.0411
Pair 6	0.0348
**Average**	0.0372
